# Providing lipid-based nutrient supplement during pregnancy does not reduce the risk of maternal *P falciparum* parasitaemia and reproductive tract infections: a randomised controlled trial

**DOI:** 10.1186/s12884-016-1215-2

**Published:** 2017-01-17

**Authors:** Minyanga Nkhoma, Per Ashorn, Ulla Ashorn, Kathryn G. Dewey, Austrida Gondwe, John Mbotwa, Stephen Rogerson, Steve M. Taylor, Kenneth Maleta

**Affiliations:** 1 University of Tampere School of Medicine, University of Tampere, Arvo building, FI-33014 Tampere, Finland; 2 Department of Pediatrics, Tampere University Hospital, FI-33521 Tampere, Finland; 3 Department of Nutrition, University of California, One Shields Ave., Davis, CA 95616-8669 USA; 4 School of Public Health and Family Medicine, College of Medicine, Mahatma Gandhi Road, Blantyre, Malawi; 5 Department of Medicine at the Peter Doherty Institute, 792 Elizabeth Street, Melbourne, VIC 3000 Australia; 6 Division of Infectious Diseases and International Health, Duke University Medical Center, Box 102359 DUMC, Durham, NC 27705 USA; 7 Department of Epidemiology, University of North Carolina, Chapel Hill, NC 27599-7435 USA

**Keywords:** Micronutrients, Pregnancy, *Plasmodium falciparum*, Reproductive tract infections, Urinary tract infection

## Abstract

**Background:**

Maternal infections are associated with maternal and foetal adverse outcomes. Nutrient supplementation during pregnancy may reduce the occurrence of infections by improving maternal immunity. We aimed to investigate the impact of small-quantity lipid-based nutrient supplement (SQ-LNS) on the occurrence of *Plasmodium falciparum* parasitaemia during pregnancy and trichomoniasis, vaginal candidiasis and urinary tract infection (UTI) after delivery.

**Methods:**

Pregnant Malawian women enrolled in the iLiNS-DYAD trial receiving daily supplementation with SQ-LNS, multiple micronutrients (MMN) or iron & folic acid (IFA) from <20 gestation weeks (gw) were assessed for *P. falciparum* parasitaemia at 32 gw using rapid diagnostic testing (RDT), at 36 gw using polymerase chain reaction (PCR) and at delivery using both RDT and PCR; and at one week after delivery for trichomoniasis and vaginal candidiasis using wet mount microscopy and for UTI using urine dipstick analysis. The prevalence of each infection by intervention group was estimated at the prescribed time points and the global null hypothesis was tested using logistic regression. Adjusted analyses were performed using preselected covariates.

**Results:**

The prevalence of *P. falciparum* parasitaemia was 10.7% at 32 gw, 9% at 36 gw, and 8.3% by RDT and 20.2% by PCR at delivery. After delivery the prevalence of trichomoniasis was 10.5%, vaginal candidiasis was 0.5%, and UTI was 3.1%. There were no differences between intervention groups in the prevalence of any of the infections.

**Conclusion:**

In this population, SQ-LNS did not influence the occurrence of maternal *P. falciparum* parasitaemia, trichomoniasis, vaginal candidiasis or UTI.

**Trial registration:**

Identifier: NCT01239693 (10 November 2010).

**Electronic supplementary material:**

The online version of this article (doi:10.1186/s12884-016-1215-2) contains supplementary material, which is available to authorized users.

## Background

Between 1990 and 2011 it was estimated that about 32% of pregnant women attending antenatal clinics in East and Southern Africa had peripheral malaria parasitaemia [1]. Most of these infections, which are usually asymptomatic, are caused by *Plasmodium falciparum* and may lead to maternal anaemia [2] and low birth weight (LBW) [3, 4]. Reproductive tract infections (RTIs) caused by *Trichomonas vaginalis* and *Candida albicans* and urinary tract infection (UTI) are also very common among pregnant women in this region and they have been associated with the occurrence of preterm birth [4–9]. LBW and preterm birth have adverse consequences for neonatal survival, subsequent childhood mortality and impaired motor and cognitive development [10–12].

Both *P. falciparum* infection and RTIs may be modified by preventive measures or presumptive treatment during pregnancy [13, 14]. For the prevention of malaria infection in pregnancy, the World Health Organisation recommends the use of long-lasting insecticidal nets (LLINs) and in areas of stable transmission in sub-Saharan Africa, intermittent preventive treatment in pregnancy with sulphadoxine pyrimethamine (IPT-SP) [15]. While there are no recommendations for the prevention of RTIs during pregnancy, programs that screen and treat RTIs early in pregnancy have been associated with a decline in the occurrence of preterm birth and LBW [16]. Nevertheless, these prevention approaches have their own challenges including vector resistance to pyrethroid, the main insecticide used in malaria control [17]; *P. falciparum* resistance to SP [18]; and the risk of the development of widespread antibiotic resistance by bacterial organisms [19] if routine antibiotic use for the prevention of RTIs was adopted. For these reasons alternative methods for the prevention of maternal infections such as nutritional interventions are sought. Provision of small-quantity lipid-based nutrient supplements (SQ-LNS) is a novel nutritional intervention that supplies multiple micronutrients (MMN) and some key macronutrients such as essential fatty acids (EFAs) embedded in a lipid base [20]. When provided during pregnancy, SQ-LNS have been shown to improve foetal growth in Ghana [21] and Bangladesh [22] and birth length in Burkina Faso [23]. However, no study has looked at the impact of antenatal provision of SQ-LNS on maternal infections.

To investigate the impact of SQ-LNS on maternal infections we measured the prevalence of maternal *P. falciparum* parasitaemia during pregnancy and at delivery and the prevalence of RTIs (trichomoniasis and vaginal candidiasis) and bacterial UTI after delivery among women in Mangochi, Malawi who were enrolled into a randomised, controlled trial that provided iron and folic acid (IFA), MMN or SQ-LNS daily [24]. We hypothesized that gestational SQ-LNS supplementation would reduce the prevalence of *P. falciparum* parasitaemia during pregnancy and the prevalence of RTIs after delivery compared to MMN and IFA. We based our assumptions on the knowledge that derivatives of the EFAs such as eicosapentaenoic acid, docosahexaenoic acid and arachidonic acid have antimalarial, antitrichomonal and antifungal properties [25–27].

## Methods

The study methods have been described in detail elsewhere [24]. Briefly, this study was a sub-study of the iLiNS-DYAD-M trial, an outcome assessor-blinded randomised controlled trial that provided antenatal nutrient supplementation to improve pregnancy outcomes and child growth (trial registration: www.clinicaltrials.gov, trial identification NCT01239693). We recruited the study participants from antenatal clinics at four health facilities in Mangochi District, southern Malawi. Pregnant women were eligible to participate in the study if they were at least 15 years old, had ultrasound-confirmed pregnancy of <20 gestation weeks (gw), had no chronic medical condition or allergies and no evident pregnancy complications. The catchment population was rural to semi-urban and subsisted mainly on fishing and farming. Maize was the staple food in this population, which faces seasonal food insecurity with a lean period just before harvest time. Malaria transmission was holoendemic in this area.

The enrolled women were randomised into three intervention groups receiving either one tablet of IFA, the standard antenatal nutrient supplement in Malawi; one tablet of MMN which contained 18 micronutrients (including iron and folic acid) or one 20 g sachet of SQ-LNS containing the same 18 micronutrients and four additional minerals, EFAs (linoleic acid and α-linolenic acid), protein, fat and 1.2 g of sucrose daily from enrolment to delivery [24]. The dose of iron was higher for women in the IFA group (60 mg) than for those in the MMN and SQ-LNS groups (20 mg). The lower iron dose in SQ-LNS was chosen so as not to greatly exceed the recommended daily intake of iron for lactating women [28], and because a dose of 20 mg/day has in some conditions been sufficient for preventing iron deficiency anaemia in pregnancy [20, 29]. The doses of the rest of the micronutrients in the MMN and SQ-LNS supplements were at least the recommended dietary allowance (RDA) for pregnancy, or the maximum amount that could be included in the supplement considering technical constraints [20]. The exact ingredients and dosing of the study supplements are shown in Additional file 1: Table S1. The participants also received two doses of IPT-SP according to the Malawi national guidelines at the time, which were concordant with the World Health Organisation’s recommendations [30].

The outcomes reported here were pre-specified secondary outcomes measures. These were peripheral *P. falciparum* parasitaemia; trichomoniasis; vaginal candidiasis; and bacterial UTI. We diagnosed *P. falciparum* parasitaemia by rapid diagnostic testing (RDT) at 32 gw, by polymerase chain reaction (PCR) at 36 gw and by RDT and PCR at delivery. RDT at 32 gw was assessed from finger prick blood samples whereas PCR at 36 gw and RDT and PCR at delivery were assessed from venous blood samples. Study nurses performed RDT according to the manufacturer’s instructions using Clearview® Malaria Combo (British Biocell International Ltd., Dundee, UK) which detects the antigens histidine-rich protein 2 and Plasmodium-specific aldolase. We conducted PCR testing for *P. falciparum* on dried blood spot samples collected from whole blood, which were tested in a real-time PCR assay targeting the *P. falciparum* lactate dehydrogenase (*pfldh*) gene [31].

RTIs and UTIs were assessed at one week after delivery using samples from vaginal swabs and mid-stream urine, respectively. The study participant was lying in the dorsal lithotomy position for the collection of the vaginal samples. After digitally separating the labia, a study nurse advanced a cotton tipped swab through the vaginal introitus towards the posterior fornix and rotated the sample back and forth three times before pulling it out. Then the vaginal samples were sent to the laboratory where a laboratory technician processed them immediately by smearing the vaginal discharge from the swab on a microscope slide and performed wet mount microscopy after adding saline solution. We defined trichomoniasis as the visualisation of motile protozoa and candidiasis as the observation of budding yeasts, pseudohyphae or hyphal forms on microscopy. The study nurse performed urinalysis immediately after urine collection using Multistix ® 10 SG reagent strip (Siemens Medical Solutions USA, Inc., Pennsylvania, USA) and UTI was indicated by the presence of nitrite in a freshly voided urine sample [32].

A sample size of 1400 was originally calculated in accordance with the main objectives of the iLiNS-DYAD-M trial. This sample size provided 80% power and 95% confidence to detect an approximately 30% reduction (among the women who received SQ-LNS, as compared to the control women) in the prevalence of maternal RTIs (from 25 to 18.3%) or *P. falciparum* parasitaemia (from 15 to 9.5%). We conducted statistical analyses using Stata 12.1 (StataCorp, College Station, USA), according to an analysis plan written and published before the intervention code was opened [24]. The analysis was based on the principle of intention-to-treat.

We estimated the prevalence of maternal *P. falciparum* parasitaemia (by intervention group) at 32 gw, 36 gw and delivery and we estimated the prevalence of the RTIs (by intervention group) at one week after delivery. We calculated Risk Ratios (RRs) for the comparison of binary end-points at a single time point and computed 95% confidence intervals (CIs) for all the effect estimates.

We performed tests for interaction between the intervention, maternal infection and pre-specified covariates using the likelihood ratio test. The pre-specified covariates were maternal age, maternal parity, gestational age at enrolment, socio-economic score, education (completed years at school) and body-mass-index (BMI) as continuous variables; HIV status, *P. falciparum* parasitaemia, anaemia, season at enrolment and study site as categorical variables. We performed stratified analyses for all covariates that had a statistically significant interaction (*p* < 0.1).

The study protocol was approved by the College of Medicine Research and Ethics Committee, University of Malawi and the Ethics Committee of Pirkanmaa Hospital District, Finland. The trial was performed according to Good Clinical Practice guidelines and the ethical standards of Helsinki Declaration. Study staff obtained informed consent from the study participants (or their legal guardian in the case of minors) prior to performing any study procedures. An independent data safety and monitoring board monitored the incidence of suspected serious adverse events and performed interim analyses for safety.

## Results

We recruited a total of 1391 pregnant women into the iLiNS-DYAD-M study between February 2011 and August 2012. The last delivery occurred in February 2013. The three groups were similar at baseline in terms of the participants’ average demographic and socioeconomic characteristics and nutritional and health status (*p* > 0.05) (Table 1). The overall rate of usage of LLINs was 70.9% and was similar across the groups (*P* = 0.536). We excluded 12 women from the analysis because they had twin pregnancies.

**Table 1:** Baseline characteristics of the participating women at enrolment, by intervention group

Characteristic	IFA	MMN	LNS
Number of participants	463	466	462
Mean (SD) maternal age, years	25 (6)	25 (6)	25 (6)
Mean (SD) maternal education, completed years	3.9 (3.4)	4.1 (3.4)	4.1 (3.6)
Proportion with severely food insecure households	34.7%	37.5%	35.8%
Mean (SD) gestational age at enrolment, weeks	16.8 (2.1)	16.8 (2.2)	16.9 (2.2)
Mean (SD) number of previous pregnancies	2.1 (1.8)	2.1 (1.8)	2.2 (1.7)
Proportion of nulliparous women	20.4%	23.0%	22.1%
Mean (SD) height, cm	156.1 (5.7)	156.0 (5.6)	156.1 (5.7)
Mean (SD) weight, kg	53.9 (7.4)	54.0 (8.1)	54.3 (8.4)
Mean (SD) MUAC, cm	26.4 (2.4)	26.3 (2.8)	26.5 (2.7)
Mean (SD) BMI, kg/m^2^	22.1 (2.6)	22.2 (2.9)	22.2 (3.0)
Proportion of women with a BMI < 18.5 kg/m^2^	5.9%	4.6%	5.7%
Mean (SD) blood haemoglobin concentration, g/l	111 (17)	111 (16)	112 (16)
Proportion of anaemic women (Hb < 100 g/l)	21.0%	19.8%	21.2%
Proportion of women with a positive HIV test	15.6%	11.1%	14.4%
Proportion of women with a positive P. falciparum test (RDT)	22.7%	24.1%	22.8%

*IFA* iron and folic acid, *LNS* lipid based nutrient supplement, *MMN* multiple micronutrients, *MUAC* mid-upper arm circumference, *RDT* rapid diagnostic test

**Fig. 1:**
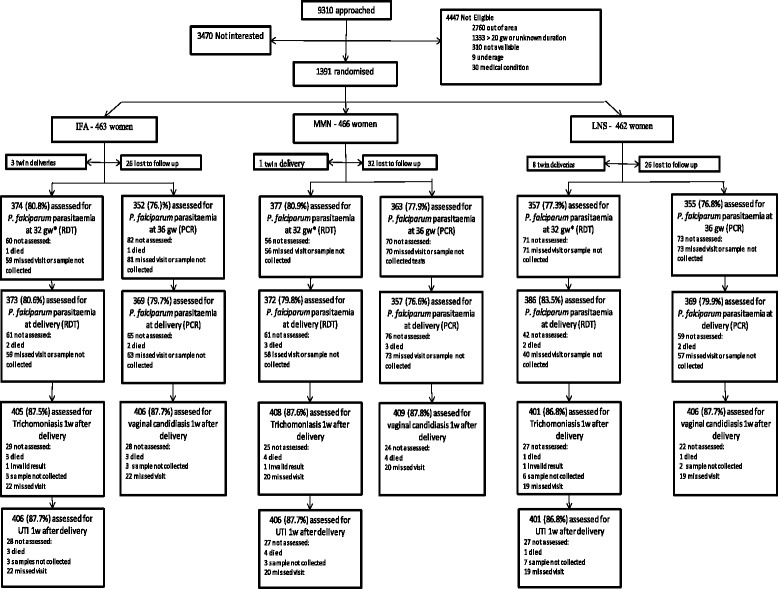
Study participant flow diagram

Follow up data for *P. falciparum* parasitaemia were available from 79.6% of the women at 32 gw, 76.9% at 36 gw, and from 81.4 and 78.7% at delivery by RDT and PCR respectively. Loss to follow up was similar between the groups (*P* = 0.805 at 32 gw, 0.638 at 36 gw, 0.180 and 0.393 for *P. falciparum* parasitaemia RDT and PCR at delivery). RTI and UTI data were collected from 86.6% of the women. Loss to follow up was similar between the groups (*P* = 0.958 for RTI and UTI after delivery) (Fig. 1). The participants who were excluded from the analysis tended to be younger (23.8 vs 25.4, *P* < 0.001); to be more educated (4.4 vs 3.9, *P* = 0.025); to have a slightly higher BMI (22.6 vs 22.0, *P* = 0.001); to have a higher wealth index (0.29 vs −0.09, *P* < 0.001); to be primiparous (31% vs 18%, *P* < 0.001) and to be anaemic (25% vs 19%, *P* = 0.014).

The overall prevalence of *P. falciparum* parasitaemia was 23.3% at enrolment (RDT), 10.7% at 32 gw (RDT); 9.0% at 36 gestation weeks (PCR); 8.3% by RDT at delivery and 20.2% by PCR at delivery. The prevalence of *P. falciparum* parasitaemia at 32 gw (RDT), 36 gw (PCR), delivery (RDT) and delivery (PCR) was 10.9, 7.6, 8.0 and 17.6%, respectively, in the LNS group, and was similar in the IFA and MMN groups (Table 2). Relative to the IFA group, the risk for *P. falciparum* parasitaemia.at 32 gw (RDT) was not significantly different in the SQ-LNS (RR 0.91, 95% CI 0.6- 1.36) or MMN (RR 0.79, 95% CI 0.52- 1.20) groups. Similarly, when compared to MMN, the risk for *P. falciparum* parasitaemia at 32 gw (RDT) in the LNS group was not significantly different (RR 1.14, 95% CI 0.74- 1.76). The risk for *P. falciparum* parasitaemia at the other time points was also similar in all 3 study groups (Table 2).

**Table 2:** Prevalence of maternal *P. falciparum* parasitaemia in Malawian women given IFA, MMN and LNS^a^

Outcome	IFA	MMN	LNS	LNS vs. IFA	LNS vs. MMN	MMN vs. IFA
*P. falciparum* parasitaemia at 32 gw (RDT)	12.0 (8.7–15.3) (374)^a^	9.5 (6.6–12.5) (377)	10.9 ( 7.7–14.2) (357)	0.91 (0.61–1.36)	1.14 (0.74–1.76)	0.79 (0.52–1.20)
*P. falciparum* parasitaemia at 36 gw (PCR)	10.8 (7.5–14.1) (352)	8.5 (5.6–11.4) (363)	7.6 (4.8–10.4) (355)	0.70 (0.44–1.13)	0.89 (0.54–1.46)	0.79 (0.50–1.24)
*P. falciparum* parasitaemia at delivery (RDT)	9.1 (6.2–12.0) (373)	7.8 (5.1–10.5) (372)	8.0 (5.3–10.8) (386)	0.88 (0.55–1.40)	1.03 (0.63–1.67)	0.86 (0.53–1.37)
*P. falciparum* parasitaemia at delivery (PCR)	20.6 (16.5–24.7) (369)	22.4 (18.0–26.8) (357)	17.6 (13.7–21.5) (369)	0.86 (0.63–1.15)	0.79 (0.59–1.05	1.09 (0.82–1.44)

^a^Values are percentages (95% CIs) (n) or risk ratios (95% CIs). *IFA* iron folic acid, *MMN* multiple micronutrients, *LNS* lipid based nutrient supplements, *RDT* rapid diagnostic testing, *PCR* polymerase chain reaction

The overall prevalence of trichomoniasis, candidiasis and UTI after delivery was 10.5, 0.5 and 3.1% respectively. The prevalence of trichomoniasis, candidiasis and UTI was 8.3, 0.3, 3.2% respectively, in the LNS group, and was similar in the IFA and MMN groups (Table 3). Relative to the IFA group, the risk for trichomoniasis was not significantly different in the SQ-LNS (RR 0.71, 95% CI 0.47- 1.09) or MMN (RR 0.93, 95% CI 0.63- 1.37) groups. Similarly, when compared to MMN, the risk for trichomoniasis in the LNS group was not significantly different (RR 0.77, 95% CI 0.50- 1.18). The risk for UTI was also similar in all 3 study groups (Table 3). The risk ratio for candidiasis was not calculated because the prevalence of candidiasis was very low.

**Table 3:** Prevalence of reproductive tract infections after delivery in Malawian women given IFA, MMN and LNS^a^

Outcome	IFA	MMN	LNS	LNS vs. IFA	LNS vs. MMN	MMN vs. IFA
Prevalence of trichomoniasis	11.6 (8.5–14.7) (405)^a^	10.8 (7.8–13.8) (407)	8.3 (5.6–11.0) (398)	0.71 (0.47–1.09)	0.77 (0.50–1.18)	0.93 (0.63–1.37)
Prevalence of candidiasis	0.2 (−0.2–0.7%) (406)	1.0 (0.0–1.9) (409)	0.3 (−0.2–0.7) (399)	–	–	–
Prevalence of UTI	2.2 (0.7–3.7) (406)	3.7 (1.9–5.6) (405)	3.2 (1.5–5.0) (401)	1.46 (0.63–3.38)	0.86 (0.42–1.82)	1.67 (0.74–3.77)

^a^Values are percentages (95% CIs) (n) or risk ratios (95% CIs). *IFA* iron folic acid, *MMN* multiple micronutrients, *LNS* lipid based nutrient supplements, *UTI* urinary tract infection

Tests of interaction with potential effect modifiers were not significant (*P* > 0.1) for maternal age, maternal parity, socio-economic score, HIV status, anaemia, season at enrolment and study site, but were significant for at least one outcome for gestational age at enrolment, maternal BMI, maternal education and baseline *P. falciparum* parasitaemia. Gestational age at enrolment modified the association between the intervention group and *P. falciparum* parasitaemia by RDT at 32 gw (*P* = 0.024); maternal BMI modified the association between the intervention group and trichomoniasis (*P* = 0.039): maternal education modified the association between the intervention group and *P. falciparum* by RDT at delivery (*P* = 0.048) and baseline *P. falciparum* parasitaemia modified the association between the intervention group and *P. falciparum* parasitaemia at delivery (by both RDT and PCR, *P* = 0.058 and 0.078 respectively). However, there were no statistically significant differences between the intervention groups in any of the strata (Additional file 2: Table S2).

## Discussion

We investigated whether the provision of SQ-LNS during pregnancy affects the occurrence of *P. falciparum* parasitaemia and RTIs among women in a population with generally poor food security. Overall, we found no statistically significant differences in the prevalence of maternal *P. falciparum* parasitaemia and RTIs between the study intervention groups.

The probabilities of bias or random error in this study were minimised by the large sample size, randomised study design, blinding of outcome assessors and inclusion of several *P. falciparum* parasitemia measurements using sensitive detection methods (RDT and PCR) [31, 33]. The success of follow-up was not complete, due to loss of participants during the follow up or missed visits or biological samples, but the proportion of missing data was similar across the study groups and several adjusted analyses gave results similar to the main analysis; hence we believe that the missing data did not significantly bias our conclusions. The excluded participants were, however somewhat different from those included in the analysis in terms of some of their anthropometric, sociodemographic and health characteristics. For these reasons our results may not be fully representative of the target population. Furthermore, the methods that we used to detect vaginal candidiasis, trichomoniasis and bacterial UTI have low to moderate sensitivity [34–36]. This may have led us to underestimate the burden of these conditions in our study population. Finally, the prevalence of each infection (except for *P. falciparum* parasitaemia at 36gw by PCR) was lower than what we had estimated in our sample size calculation, potentially affecting our power calculations. However, as argued by Feinstein and Concato [37], our point estimates and confidence intervals were consistently in the same direction and sufficiently precise to conclude that SQ-LNS supplementation did not influence the prevalence of maternal *P. falciparum* parasitemia or RTIs in rural Malawi, though we cannot conclusively rule out an effect on the prevalence of candidiasis and UTI.

The prevalence of *P. falciparum* parasitemia at delivery was much higher based on PCR testing compared to RDT among the study women. This finding was not unusual and it was probably due to low parasite density in the study population. The sensitivity of RDT tests varies with parasite density, with lower sensitivity at lower parasite density [38].

The prevalence of *P. falciparum* parasitaemia by RDT continued to decline from enrolment through 32 gestational weeks to delivery in our study population. This finding is unsurprising as similar results were obtained from women who received two doses of IPT-SP in a previous study conducted in the same study area [14] and from elsewhere in sub-Saharan Africa [39]. Assuming no loss to follow-up in our study, we would expect the prevalence of *P. falciparum* parasitaemia in pregnancy to drop in the period following enrolment into the study as the women were exposed to anti-malaria interventions such as IPT-SP and receipt of LLINs as part of the ante-natal care package.

Little is known about the effect of SQ-LNS on maternal health or morbidity. Other reports from the iLiNS-DYAD trial suggested that SQ-LNS and MMN may have increased the risk of maternal dental infections [40] on one hand but seemed to have had no effect on the development of maternal anti-malarial antibodies [41] on the other hand. Previous in vitro studies have suggested that EFAs and their derivatives such as arachidonic acid and docosahexaenoic acid may have toxic effects on *P. falciparum* [42], *T. vaginalis* [43] and *C. albicans* [27] via different mechanisms*,* hence our expectation that providing SQ-LNS which contained EFAs would reduce the prevalence of infections caused by these organisms. The apparent lack of benefit from the EFAs in this study could have happened because the study women were not deficient in EFAs to begin with. A recently published trial conducted in the same study area as our study found that the levels of breast milk arachidonic acid and docosahexaenoic acid were above average levels [44] contrary to our earlier assumptions based on previous studies in sub-Saharan Africa [45]. Alternatively, the absence of influence of SQ-LNS on maternal plasma composition of EFAs (Oaks et al., in press) could explain our observation of lack of impact.

A previous study that provided combined vitamin A and zinc supplementation to children in Ghana led to a reduction in the risk of clinical malaria episodes [46] possibly due to the synergistic effects of vitamin A and zinc on immune function [47]. Contrary to our expectation based on these previous studies, SQ-LNS and MMN which contained both vitamin A and zinc were not associated with a lower risk of *P. falciparum* parasitaemia. The lack of treatment response despite the provision of adequate doses of vitamin A and zinc in MMN and SQ-LNS and reasonable adherence to supplementation [24] could have been due to dietary factors inhibiting zinc absorption [48] or haemodilution in the latter half of pregnancy lowering levels of retinol binding protein resulting in functional vitamin A deficiency [49].

## Conclusion

Taken together, the study findings do not support our hypothesis that the provision of antenatal SQ-LNS would reduce the prevalence of *P. falciparum* parasitaemia during pregnancy and the prevalence of RTIs after delivery compared to supplementation with MMN or IFA. Whether or not a target population is deficient in EFAs might be an important factor in determining whether SQ-LNS provision to pregnant women can influence the occurrence of maternal infections. Use of more sensitive diagnostic methods such as culture, antigen or PCR tests for the detection of RTIs and UTI may improve outcome ascertainment in future studies. Further clinical trials supplementing single nutrients (EFAs, vitamin A or zinc) may be crucial in isolating the impact of each nutrient on maternal infections.

## Additional files

Additional file 1: Table S1. Nutrient contents of the dietary supplements provided in the iLiNS-DYAD-M Trial. (DOCX 17 kb)
Additional file 2: Table S2. Effect Modification – Malaria Parasitaemia and Trichomoniasis by Intervention Group, Stratified Analysis. (DOCX 19 kb)

## Abbreviations

BMI: Body-mass-index
CIs: Confidence Intervals
EFAs: Essential fatty acids
gw: Gestation weeks
IFA: Iron and folic acid
IPT-SP: Intermittent preventive treatment with sulphadoxine pyrimethamine
LBW: Low birth weight
LLINs: Long-lasting insecticide treated nets
MMN: Multiple micronutrients
PCR: Polymerase chain reaction
RDT: Rapid diagnostic testing
RRs: Risk ratios
RTIs: Reproductive tract infections
SQ-LNS: Small-quantity lipid-based nutrient supplements
UTI: Urinary tract infection
