# Supplementary material for: Providing lipid-based nutrient supplement during pregnancy does not reduce the risk of maternal P falciparum parasitaemia and reproductive tract infections: a randomised controlled trial
Source: BMC Pregnancy Childbirth. 2017 Jan 17;17:35. doi: 10.1186/s12884-016-1215-2 (PMC5240436; doi:10.1186/s12884-016-1215-2)
Supplement: Additional file 1: Table S1. — Nutrient contents of the dietary supplements provided in the iLiNS-DYAD-M Trial. (DOCX 17 kb) [file 12884_2016_1215_MOESM1_ESM.docx]

**Table S1. Nutrient contents of the dietary supplements provided in the iLiNS-DYAD-M Trial**

| Nutrient | IFA | MMN | LNS |
| --- | --- | --- | --- |
| Ration (g/day) | 1 tablet | 1 tablet | 20 g sachet |
| Protein (g) | 0 | 0 | 2.6 |
| Fat (g) | 0 | 0 | 10 |
| Linoleic acid (g) | 0 | 0 | 4.59 |
| α-Linolenic acid (g) | 0 | 0 | 0.59 |
| Vitamin A (μg RE) | 0 | 800 | 800 |
| Vitamin C (mg) | 0 | 100 | 100 |
| Vitamin B1(mg) | 0 | 2.8 | 2.8 |
| Vitamin B2 (mg) | 0 | 2.8 | 2.8 |
| Niacin (mg) | 0 | 36 | 36 |
| Folic acid (μg) | 400 | 400 | 400 |
| Pantothenic acid (mg) | 0 | 7 | 7 |
| Vitamin B6 (mg) | 0 | 3.8 | 3.8 |
| Vitamin B12 (μg) | 0 | 5.2 | 5.2 |
| Vitamin D (IU) | 0 | 400 | 400 |
| Vitamin E (mg) | 0 | 20 | 20 |
| Vitamin K (μg) | 0 | 45 | 45 |
| Iron (mg) | 60 | 20 | 20 |
| Zinc (mg) | 0 | 30 | 30 |
| Cu (mg) | 0 | 4 | 4 |
| Calcium (mg) | 0 | 0 | 280 |
| Phosphorus (mg) | 0 | 0 | 190 |
| Potassium (mg) | 0 | 0 | 200 |
| Magnesium (mg) | 0 | 0 | 65 |
| Selenium (μg) | 0 | 130 | 130 |
| Iodine (μg) | 0 | 250 | 250 |
| Manganese (mg) | 0 | 2.6 | 2.6 |

IFA, iron folic acid; MMN, multiple micronutrients and LNS, lipid based nutrient supplements
