# Supplementary material for: Providing lipid-based nutrient supplement during pregnancy does not reduce the risk of maternal P falciparum parasitaemia and reproductive tract infections: a randomised controlled trial
Source: BMC Pregnancy Childbirth. 2017 Jan 17;17:35. doi: 10.1186/s12884-016-1215-2 (PMC5240436; doi:10.1186/s12884-016-1215-2)
Supplement: Additional file 2: Table S2. — Effect Modification – Malaria Parasitaemia and Trichomoniasis by Intervention Group, Stratified Analysis. (DOCX 19 kb) [file 12884_2016_1215_MOESM2_ESM.docx]

**Table S2.** Effect Modification – Malaria Parasitaemia and Trichomoniasis by Intervention Group, Stratified Analysis

| **Outcome** | **Interaction test P-value** | **Result by intervention group** | | | | **Comparison between LNS group and IFA group** | | **Comparison between LNS group and MMN group** | | **Comparison between MMN group and IFA group** | |
| --- | --- | --- | --- | --- | --- | --- | --- | --- | --- | --- | --- |
|  |  | **IFA** | **MMN** | **LNS** | **P-value^a^** | **Risk Ratio (95% CI)** | **P-value^b^** | **Risk Ratio (95% CI)** | **P-value^b^** | **Risk Ratio (95% CI)** | **P-value^b^** |
| ***Malaria Parasitaemia at 32 gw (RDT)*** | | | | | | | | | | | |
| Gestation age at enrollment <16.9  (weeks) | 0.024 | 24/184  (13.0%) | 11/182  (6.0%) | 17/169  (10.1%) | 0.320 | 0.77 (0.43 to 1.38) | 0.384 | 1.66 (0.80 to 3.45) | 0.171 | 0.46 (0.23 to 0.92) | 0.027 |
| Gestation age at enrollment ≥16.9 (weeks) |  | 21/190  (11.0%) | 25/195  12.8% | 22/188  (11.7%) | 0.844 | 1.06 (0.60 to 1.86) | 0.842 | 0.91 (0.53 to 1.56) | 0.739 | 1.16 (0.67 to 2.00) | 0.593 |
| ***Malaria Parasitaemia at Delivery (RDT)*** | | | | | | | | | | | |
| Maternal educational achievement <4 years | 0.048 | 16/187  (8.6%) | 20/170  (11.8%) | 14/190  (7.4%) | 0.685 | 0.86 (0.43 to 1.71) | 0.670 | 0.63 (0.33to 1.20) | 0.159 | 1.38 (0.74 to 2.57) | 0.317 |
| Maternal educational achievement ≥4 years |  | 18/186  (9.7%) | 9/202  (4.5%) | 17/196  (8.7%) | 0.734 | 0.90 (0.48 to 1.69) | 0.734 | 1.95 (0.89 to 4.26) | 0.096 | 0.46 (0.21 to 1.00) | 0.050 |
| ***Malaria Parasitaemia at Delivery (RDT)*** |  |  |  |  |  |  |  |  |  |  |  |
| No malaria (RDT) at enrollment | 0.058 | 28/299  (9.4%) | 15/284  (5.3%) | 22/297  (7.4%) | 0.360 | 0.79 (0.46 to 1.35) | 0.390 | 1.40 (0.74 to 2.65) | 0.297 | 0.56 (0.31 to 1.03) | 0.064 |
| Malaria (RDT) at enrollment |  | 6/74  (8.1%) | 14/87  (16.1%) | 9/87  (10.3%) | 0.716 | 1.28 (0.48 to 3.42) | 0.628 | 0.64 (0.29to 1.41) | 0.269 | 1.98 (0.80 to 4.90) | 0.138 |
| ***Maternal Parasitaemia At Delivery (PCR)*** | | | | | | | | | | | |
| No malaria (RDT) at enrollment | 0.078 | 59/288 (20.5%) | 51/272  (18.8%) | 50/278  (18.0%) | 0.448 | 0.88 (0.63 to 1.23) | 0.451 | 0.96 (0.67 to 1.36) | 0.817 | 0.92 (0.65 to 1.28) | 0.606 |
| Malaria (RDT) at enrollment |  | 17/81 (21.0%) | 28/84 (33.3%) | 15/90 (16.7%) | 0.458 | 0.79 (0.42 to 1.49) | 0.470 | 0.50 (0.29 to 0.87) | 0.817 | 1.59(0.94 to 2.67) | 0.081 |
| ***Trichomoniasis at 1 w after Delivery*** | | | | | | | | | |  |  |
| BMI <21.6 | 0.039 | 26/204 (12.8%) | 27/201 (13.4%) | 14/190 (7.4%) | 0.098 | 0.58 (0.31 to 1.07) | 0.116 | 0.55 (0.30 to 1.01) | 0.079 |  |  |
| BMI ≥21.6 |  | 21/201 (10.5%) | 17/206 (8.3%) | 19/208 (9.1%) | 0.652 | 0.87 (0.48 to 1.58) | 0.655 | 1.02 (0.59 to 2.07) | 0.750 |  |  |
